# CanLRHI: a multimodal pretraining model for cell death analysis in cancer pathology based on long-text representation and high-resolution images

**DOI:** 10.1093/bib/bbag341

**Published:** 2026-06-22

**Authors:** Tianjiao Zhang, Long Wan, Hongfei Zhang, Zhongqian Zhao, Haijie Cui, Jianli Ma

**Affiliations:** The School of Computer Science and Artificial Intelligence, Northeast Forestry University, 26 Hexing Road, Xiangfang District, Harbin, Heilongjiang 150040, China; The School of Computer Science and Artificial Intelligence, Northeast Forestry University, 26 Hexing Road, Xiangfang District, Harbin, Heilongjiang 150040, China; The School of Computer Science and Artificial Intelligence, Northeast Forestry University, 26 Hexing Road, Xiangfang District, Harbin, Heilongjiang 150040, China; The School of Computer Science and Artificial Intelligence, Northeast Forestry University, 26 Hexing Road, Xiangfang District, Harbin, Heilongjiang 150040, China; The Department of Radiation Oncology, Harbin Medical University Cancer Hospital, 150 Haping Road, Nangang District, Harbin, Heilongjiang 150081, China; The Department of Radiation Oncology, Harbin Medical University Cancer Hospital, 150 Haping Road, Nangang District, Harbin, Heilongjiang 150081, China

**Keywords:** cancer pathology, long-text, multimodal pretraining, cell death, cancer region identification

## Abstract

The high heterogeneity of cancer poses significant challenges for precision diagnosis, particularly in tasks such as rare subtype identification, early lesion detection, and tumor grading. Notably, cancer cell biological traits are closely correlated with cell death regulatory mechanisms, and accurate cancer region identification is a pivotal premise for exploring the cancer-cell death intrinsic association. Single-modality methods often struggle to balance sensitivity and accuracy, while existing general multimodal models are poorly adapted to the processing of pathological long texts and high-resolution images, leading to issues of semantic truncation and feature loss. To address these challenges, this study proposes CanLRHI, a multimodal pretraining model tailored for cancer pathology that focuses on the synergistic modeling of long pathological reports and high-resolution images to achieve comprehensive cross-modal alignment and accurate characterization of cancer regions. Experimental results on the CancerPath-170 K-v1 dataset, which contains 170 000 cancer pathology image–text pairs, demonstrate that CanLRHI significantly outperforms mainstream multimodal baselines across various tasks, including Zero-Shot classification and Few-Shot Fine-Tuning. This work provides an extensible technical framework for long-text-driven cross-modal representation learning in medical pathology, and further offers a reliable technical support for cell death-related cancer pathology research via high-precision cancer region detection.

## Introduction

In clinical pathology, diagnostic evaluation typically relies on two complementary modalities [1]. Specifically, clinical medical images (including pathological slides [2], radiological scans, and ultrasound) visually capture the morphological and structural characteristics of tumor cells. Corresponding diagnostic reports [3] record key clinical indicators such as tumor type, grade, and invasion extent.

In recent years, cross-modal representation learning has achieved substantial progress in both computer vision and natural language processing, driven by the emergence of large-scale vision-language pretraining frameworks such as Contrastive Language-Image Pre-Training (CLIP) [4], ALBEF [5], and BLIP [6]. These models, which leverage contrastive learning on massive image-text pairs, have demonstrated remarkable cross-domain generalization and adaptability to diverse downstream tasks. Despite their success in natural image understanding, their direct application to medical domains remains challenging. Pathology reports are typically long and information-dense, often exceeding the token length constraints of CLIP-like architectures, thereby causing semantic truncation and the omission of critical diagnostic cues. Furthermore, most visual encoders in these general-purpose frameworks are optimized for natural images and fail to effectively capture the fine-grained morphological and structural patterns inherent in high-resolution medical images. Consequently, such limitations hinder the robustness, interpretability, and diagnostic precision of current multimodal models in cancer pathology analysis.

To address the aforementioned challenges, existing studies have attempted to develop medical-specific multimodal models. For instance, MedCLIP [7] and BART-based approaches [8] enhance model adaptability by conducting additional pre-training on medical data or incorporating knowledge bases. However, such methods generally still overlook the full utilization of long-text information. In fact, long-text pathological descriptions not only provide conclusive diagnostic results but also retain key information such as contextual reasoning, lesion localization, and histological background. If the model relies solely on short texts or truncated report fragments, it often fails to capture the complete diagnostic logic, thereby limiting its performance in complex lesion identification and rare subtype modeling. Therefore, how to effectively adapt to medical long texts in cross-modal models has become a key breakthrough for improving diagnostic performance.

It is important to clarify that Long-CLIP [9] is employed exclusively on the textual side in this work, because the core innovation of CanLRHI lies in addressing the critical challenge of processing long pathological reports that far exceed the 77-token limit of standard CLIP. Specifically, we introduce a hierarchical text processing pipeline that integrates Cancer-BART compression, OncoCache intermediate semantic caching, and Long-CLIP encoding, enabling complete preservation of diagnostic semantics within cross-modal alignment constraints.

Moreover, this study constructs the CancerPath-170 K-v1 dataset, comprising 170 000 cancer pathology image-text pairs. Notably, over 25% of textual descriptions exceed the input length constraints of general-purpose models, thereby highlighting the imperative for long-text modeling. Experimental evaluations on this benchmark demonstrate that CanLRHI significantly outperforms state-of-the-art multimodal models across diverse tasks, including Zero-Shot classification [10, 11], Few-Shot Fine-Tuning [12], medical visual question answering (VQA) [13], and cross-modal retrieval [14]. Ablation studies further substantiate the pivotal role of the long-text compression mechanism in enhancing model performance, revealing that medical long texts not only compensate for informational deficiencies in visual modalities but also reinforce cross-modal alignment and generalization capabilities by encapsulating comprehensive diagnostic logic. Collectively, this work establishes the unique advantage of medical long-text-driven cross-modal modeling in cancer pathology diagnosis, offering an extensible technical framework for precision diagnostics and clinical decision support.

## Methods

### Dataset

From the publicly available PMC-OA [15] corpus, we extracted 1.64 million raw image-text pairs as the initial dataset, stored in JSON Lines (JSONL) format, where each record contains an image path and a paragraph-level textual caption directly obtained from the original article-suitable for subsequent filtering and preprocessing.

Since the raw corpus includes a considerable proportion of non-target visual modalities (e.g. statistical charts, flow diagrams, molecular structures), we designed a two-stage data optimization process:

In the first stage, a custom-built cancer pathology terminology lexicon was used for relevance filtering. This lexicon, developed based on authoritative oncology and pathology resources, enables precise identification of cancer-related terminology [16] such as infiltration, differentiation, adenocarcinoma, and tumor boundary. Term-based matching effectively excluded non-relevant figures (e.g. statistical plots, non-cancer disease descriptions), while retaining high-quality cancer pathology image-text pairs for multimodal preprocessing.

In the second stage, multimodal standardization was applied, including image resizing, Z-score normalization [17], and text cleaning to remove irrelevant symbols and ensure textual consistency. After rigorous screening and preprocessing, we constructed the CancerPath-170 K-v1 dataset, containing 170 000 high-quality image-text pairs, split into training and validation sets at a 9:1 ratio.

Statistically, the dataset encompasses a broad spectrum of cancer types, dominated by breast cancer (approximately 18%–22%), lung cancer (15%–18%), and colorectal cancer (10%–12%), followed by prostate cancer (8%–10%), liver cancer (6%–8%), and stomach cancer (5%–7%). Additional malignancies—including pancreatic cancer (4%–6%), ovarian cancer (3%–5%), brain tumors (3%–5%), and lymphoma (3%–5%)—are also represented, with the remaining 15%–20% covering thyroid, esophageal, cervical, and melanoma cases. Regarding image modalities, pathological images [hematoxylin and eosin (H&E) staining, immunohistochemistry (IHC) staining, etc.] constitute 20%–25% of the dataset, while clinical medical images (CT, MRI, X-ray, ultrasound) account for 40%–50%. Scientific charts and schematic diagrams comprise 15%–20%, microscopic images 10%–15%, and other medical images (anatomical diagrams, endoscopic images, etc.) 5%–10%. Regarding textual characteristics, the token length of pathological descriptions exhibits substantial variation, ranging from approximately 50 tokens to several hundred tokens, with a considerable proportion significantly exceeding the 77-token input constraint of conventional vision-language models, thereby necessitating dedicated long-text encoding mechanisms.

The dataset exhibits substantial visual and semantic diversity, covering diverse tissue types, staining protocols, and magnification scales. Furthermore, the strong semantic correlation between images and text provides a robust foundation for developing next-generation multimodal models for cancer pathology diagnosis.

### Model architecture

CanLRHI focuses on the text side, where the main challenge lies: pathology reports are typically long and information-dense, often exceeding 500 tokens, which causes severe semantic truncation in standard CLIP. To address this, the text branch employs Cancer-BART to compress long reports while preserving key diagnostic information, and then uses Long-CLIP to encode the compressed text. The image branch adjusts the Vision Transformer(ViT) [18] resolution and patch size. The two branches are aligned through symmetric contrastive learning.

**Figure 1:**
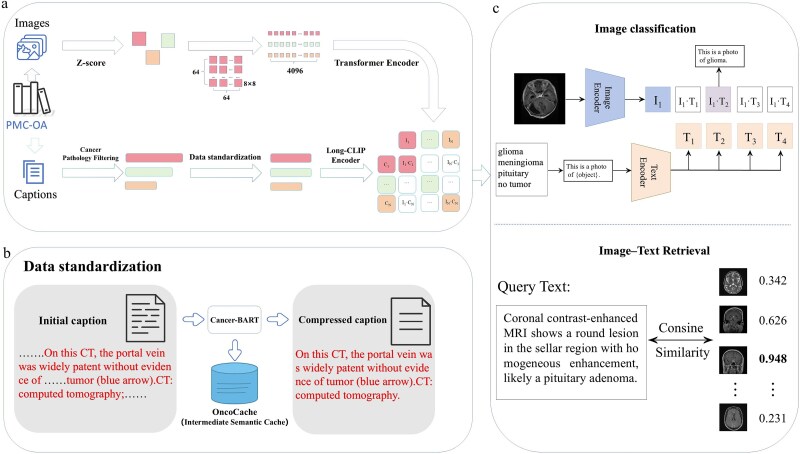
Overall framework of the CanLRHI model. (a) Data construction and encoding process: Image-text pairs are collected from the PMC-OA dataset, followed by cancer-related filtering and multimodal normalization. The text branch performs hierarchical compression and encoding to generate unified cross-modal representations, while the image branch uses an optimized vision transformer with Z-score normalization to extract fine-grained pathological features. (b) Text compression mechanism: Long pathology reports are compressed by the cancer-BART model fine-tuned on medical corpora, removing redundant background while preserving core diagnostic semantics. The compressed and original features are stored in OncoCache as an intermediate semantic layer for long-CLIP encoding and alignment. (c) Downstream tasks: The model supports image classification, image-text retrieval, and medical VQA tasks, enabling precise cross-modal alignment between high-resolution pathological images and long-form pathology reports.

As illustrated in Fig. 1, the overall architecture of CanLRHI consists of a text branch and an image branch. The text branch compresses long pathological reports through Cancer-BART and then encodes them through Long-CLIP. The image branch extracts features through a ViT with increased resolution and reduced patch size.

On the textual side, the model first compresses long pathological reports using Cancer-BART, a medical-domain fine-tuned variant of BART. The compression preserves diagnostic key information such as tumor type, grade, and invasion extent, while removing redundant background. This is not a general text summarization task, because pathological reports contain specialized entities and spatial descriptions that general-domain models tend to discard.

To bridge the compression and encoding stages, CanLRHI incorporates an intermediate semantic layer termed OncoCache, which stores the compressed representations generated by Cancer-BART prior to Long-CLIP encoding. OncoCache serves as a structured semantic buffer that preserves the integrity of compressed diagnostic information-such as lesion type, grading, and infiltration extent-while ensuring seamless handoff to the subsequent encoding stage. By decoupling the compression output from the encoder input, OncoCache prevents secondary semantic loss during format transition and enables efficient caching of frequently accessed diagnostic templates, thereby alleviating semantic truncation and stabilizing the cross-modal alignment pipeline.

In the long-text encoding stage, CanLRHI adopts the Long-CLIP architecture, which overcomes CLIP’s 77-token input limitation. This extended encoder allows full representation of compressed pathological texts, providing rich, semantically complete textual embeddings that enable robust cross-modal alignment with high-resolution image features.

On the visual side, Z-score normalization is applied during preprocessing. The visual encoder is a ViT-L/14 with input resolution increased from 224 × 224 to 512 × 512 and patch size reduced from 14 × 14 to 8 × 8.

We employ symmetric contrastive learning [19] to ensure high-precision alignment between pathological images and texts within a unified semantic space. For each image-text pair, normalized embeddings are extracted via the visual and textual encoders, and cosine similarity is used to measure correspondence. A learnable temperature parameter (τ, initialized at 0.07, consistent with the original CLIP) dynamically adjusts the smoothness of similarity distributions.

The loss function, derived from Information Noise Contrastive Estimation (InfoNCE), is computed symmetrically in both Image → Text and Text → Image directions:


(1) \begin{equation*} {L}_{I\to T}=-\frac{1}{N}\sum \limits_{i=1}^N\mathit{\log}\frac{\mathit{\exp}\left({s}_{ii}\right)}{\sum_{j=1}^N\mathit{\exp}\left({s}_{ij}\right)} \end{equation*}

(2) \begin{equation*} {L}_{T\to I}=-\frac{1}{N}\sum \limits_{i=1}^N\mathit{\log}\frac{\mathit{\exp}\left({s}_{ii}\right)}{\sum_{j=1}^N\mathit{\exp}\left({s}_{j\mathrm{i}}\right)} \end{equation*}

Final objective function is defined as the weighted average:


(3) \begin{equation*} {L}_{res}=\frac{1}{2}\left({L}_{I\to T}+{L}_{T\to I}\right) \end{equation*}

This symmetric optimization ensures bidirectional retrieval capability (image-to-text and text-to-image) in downstream tasks including retrieval, classification, and question answering, thereby enhancing the robustness and generalizability of cross-modal representations. Furthermore, the learnable temperature parameter τ adaptively modulates similarity distributions, accentuating the discriminability between positive and negative samples to optimize cross-modal semantic alignment.

### Training configuration

To ensure efficient model convergence and high-precision cross-modal alignment, the following experimental configuration was adopted:

#### Hardware environment

We conducted training on a single NVIDIA A100 GPU (80 GB memory, CUDA 11.2), which is sufficient to support large-batch training. We implemented the model using PyTorch 2.0.1.

The complete CanLRHI model contains approximately 500 million parameters. The trainable parameters consist primarily of the ViT-L visual encoder and the Long-CLIP text encoder (approximately 430 million), while the Cancer-BART compressor remains frozen during pretraining. Training was conducted on a single NVIDIA A100 GPU (80 GB memory, CUDA 11.2) with automatic mixed precision enabled. With a batch size of 80, each epoch over the 170 000 image-text pairs required approximately 1.5 hours, yielding a total training duration of approximately 8 days before the early-stopping criterion was met at epoch 132. During inference, the model processes a single image-text pair within approximately 200 milliseconds on the A100 GPU, ensuring practical feasibility for real-time clinical decision support applications.

#### Optimization strategy

Compared to the Adam optimizer, we found that SGD [20] more effectively prevents feature collapse and improves semantic alignment stability. Therefore, we employed SGD with a momentum of 0.9. We set the initial learning rate to 1 × 10^−4^ and fixed the batch size at 80, balancing convergence speed and alignment accuracy. We trained the model for 200 epochs, covering the entire training dataset.

#### Early-stopping mechanism

To prevent overfitting in later training stages and enhance generalization capability, an early-stopping strategy based on the cross-modal retrieval recall at rank 10 (R@10) metric on the validation set was applied:

Early-stopping threshold: Training stopped if the R@10 score failed to improve for five consecutive epochs;

Best model preservation: The checkpoint corresponding to the highest validation R@10 score was saved; the final early-stop occurred at epoch 132, where the validation R@10 stabilized at 0.32;

Validation protocol: 10% of the CancerPath-170 K-v1 dataset (approximately 17 000 image-text pairs) was reserved exclusively for validation, ensuring an unbiased evaluation.

### Benchmark protocol

In multimodal medical research, establishing a scientific and systematic evaluation protocol is critical for assessing model performance. Existing studies often rely on disparate datasets and task settings, resulting in inconsistent evaluation criteria that hinder fair cross-model comparison and reproducibility. To address this, we designed a standardized benchmarking framework to comprehensively evaluate the applicability and generalization capability of the proposed pretraining model across multiple pathological tasks.

This benchmark covers four representative downstream tasks, reflecting model performance across classification, transferability, reasoning, and retrieval dimensions relevant to real-world clinical applications:

#### Zero-shot classification

Pathological images are directly classified using natural-language prompts, allowing the evaluation of the model’s knowledge transferability under fully label-free conditions. This task specifically tests the adaptability of CanLRHI to low-resource scenarios, such as rare cancer subtype identification.

#### Few-shot fine-tuning

The model undergoes lightweight fine-tuning with a very limited number of labeled samples, simulating realistic medical settings where annotation resources are scarce. This task measures the model’s rapid adaptation capability and stability under constrained supervision.

#### Visual question answering

The model is required to answer diagnostic questions based on visual understanding of pathological images and corresponding question semantics. This task evaluates cross-modal reasoning, fine-grained semantic modeling, and multimodal information fusion capabilities.

#### Image–text retrieval

By performing bidirectional retrieval between images and texts, this task assesses the semantic alignment accuracy of the multimodal representation space. The results reflect the model’s practical utility in clinical applications such as case retrieval, report matching, and decision support.

Through joint evaluation across these dimensions, our benchmark not only validates model performance on individual tasks but also provides a comprehensive assessment of transferability, robustness, and real-world applicability in cancer pathology.

## Results

### Zero-shot classification

Since CanLRHI is trained through contrastive learning on paired cancer pathology images and reports, it establishes a precise intrinsic alignment between textual descriptions and visual representations. This alignment enables effective zero-shot image classification, which is particularly valuable in annotation-scarce scenarios such as rare cancer subtype recognition. To evaluate this capability, we conducted zero-shot classification experiments on several representative public cancer datasets encompassing diverse imaging modalities, including Brain Tumor MRI [21], Chest CT-Scan [22], Malignant Lymphoma Classification [23], BUSI with GT [24], and Br35H [25]. The details of these datasets, including their sizes, modalities, and corresponding class labels, are summarized in Table 1. Although the CancerPath-170 K-v1 pretraining corpus is constructed from pathology literature, the downstream evaluation deliberately includes diverse cancer imaging modalities-MRI, CT, ultrasound, and pathology slides-to comprehensively assess the model’s cross-modal generalization capability in real-world clinical scenarios. For baseline comparison, we evaluate the original CLIP model (ViT-B/32, referred to as CLIP) and its large variant (ViT-L/14, referred to as CLIP-L), both released by OpenAI, alongside other state-of-the-art methods.

**Table 1:** Overview of datasets used in the zero-shot classification experiments.

**Dataset**	**Year**	**Modality**	**Samples**	**Classes**
Brain Tumor MRI	2021	Brain MRI	7023	glioma, meningioma, pituitary, no tumor
Chest CT-Scan	2021	Chest CT (2D)	1000	adenocarcinoma, large cell carcinoma, squamous carcinoma, normal
Malignant Lymphoma Classification	2010	Pathology (2D, tif)	374	Chronic Lymphocytic Leukemia, Follicular Lymphoma, Mantle Cell Lymphoma
Dataset_BUSI_with_GT	2018	Breast Ultrasound	780	normal, benign, malignant
Br35H	2022	Brain MRI	3000	non-tumorous, tumorous

For each dataset, we designed category-specific prompts aligned with cancer pathology semantics, such as ‘This is a photo of [class].’ and ‘An H&E image of [class].’ In the shared semantic embedding space, the cosine similarity between each image and its candidate textual prompts was computed, and the class corresponding to the highest similarity score was selected as the prediction.

Here, [class] denotes the predefined diagnostic category label of each dataset (e.g. glioma, meningioma, pituitary, and no tumor for Brain Tumor MRI). It is important to emphasize that this evaluation follows a generalized zero-shot transfer protocol: although [class] represents a categorical label, CanLRHI has never been trained on any labeled samples from these downstream datasets during pre-training on CancerPath-170 K-v1. Instead, the model must infer the correct category by measuring semantic similarity between the image and candidate text prompts in the shared embedding space. To ensure a fair comparison, all baseline models-including CLIP, CLIP-L, CONCH [26], PLIP [27], and Long-CLIP-were evaluated using the identical prompt templates and category labels described above, with no additional task-specific fine-tuning or prompt engineering applied to any method. Notably, unlike few-shot learning, zero-shot classification does not involve any task-specific fine-tuning on the downstream datasets; the evaluation is inherently deterministic given the fixed pretrained weights and identical prompts across all runs. Therefore, conventional k-fold cross-validation is not applicable in this setting. All reported accuracy scores are computed on the full official test splits of each dataset to ensure reproducibility and fair comparison with baselines.

**Figure 2:**
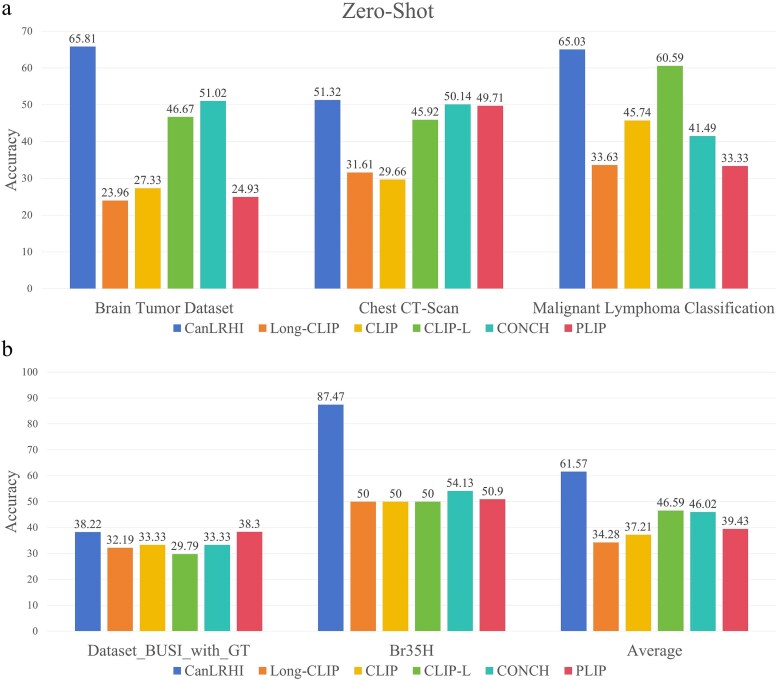
Comparison of zero-shot classification results across multiple cancer pathology datasets. (a) Accuracy comparison among CanLRHI, long-CLIP, OpenAI-CLIP, OpenAI-CLIP-L, CONCH, and PLIP on brain tumor, chest CT-scan, and malignant lymphoma datasets. (b) Results on BUSI with GT and Br35H datasets, along with the overall average across five datasets. CanLRHI achieves 87.47% accuracy on Br35H and maintains the highest mean accuracy (61.57%), significantly outperforming all baseline models.

As illustrated in Fig. 2, CanLRHI achieves a remarkable 87.47% accuracy on Br35H.On the BUSI with GT dataset, CanLRHI attains 38.22%. The relatively lower accuracy on this dataset can be attributed to several factors. First, breast ultrasound images exhibit substantially different visual textures and artifact patterns compared to the pathology slides that dominate the CancerPath-170 K-v1 pretraining corpus, resulting in a larger domain shift. Second, the three-class classification task (normal, benign, malignant) requires finer-grained discrimination of soft-tissue boundaries, which is more challenging under zero-shot transfer than the binary or coarse-grained tasks in other datasets. Third, the limited dataset size (780 images) may amplify the variance of zero-shot estimation. These observations suggest that while CanLRHI demonstrates strong cross-modal generalization, its performance on ultrasound modalities could be further improved by incorporating more diverse imaging modalities into the pretraining data or through domain-adaptive fine-tuning.

### Few-shot fine-tuning

Conventional image classification typically requires extensive annotated data to achieve high accuracy [28, 29], yet annotation resources are exceptionally scarce in medical pathology [30]. To address this limitation, we investigate CanLRHI’s performance in few-shot scenarios. Experiments were conducted on brain tumor classification and skin cancer benign-malignant classification datasets under training conditions with 1, 2, 4, 8, 16, and 32 samples per class, benchmarking against CLIP and CONCH. Each condition was replicated five times.

**Figure 3:**
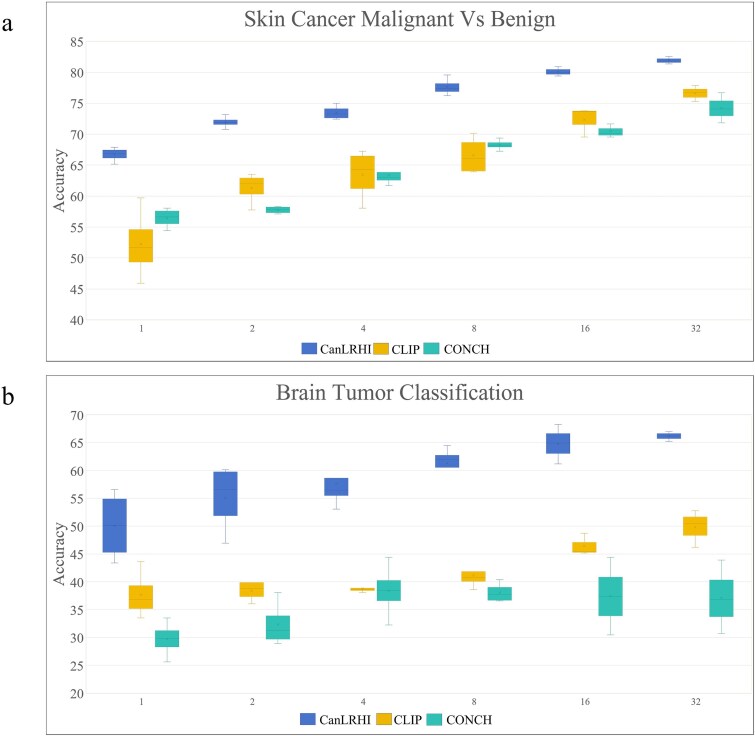
Few-shot fine-tuning results. (a) Comparison of CanLRHI, CLIP, and CONCH under different sample sizes on the skin cancer benign-malignant classification dataset. (b) Few-shot fine-tuning performance of the same models on the brain tumor pathology dataset.

As illustrated in Fig. 3, CanLRHI consistently outperforms baselines across all few-shot scales, with particularly pronounced advantages in extreme low-sample regimes (1-shot, 2-shot). Under 1-shot conditions, CanLRHI achieves an average accuracy exceeding 50%, significantly surpassing CLIP and CONCH. Performance escalates steadily with increasing sample size, exceeding 70% accuracy at 32-shot and markedly outperforming comparative models.

Notably, CanLRHI exhibits substantially lower performance variance across repeated trials compared to baselines. As the shot number increases, both accuracy and stability improve (evidenced by decreasing variance), attributable to the synergistic mechanism of long-text compression and high-resolution image representation. This synergy enables comprehensive capture of cross-modal semantics, effectively mitigating overfitting and instability in few-shot learning.

It should be emphasized that zero-shot and few-shot evaluations measure fundamentally different capabilities. Importantly, the brain tumor datasets used in these two protocols are not identical. The zero-shot experiment employs the Brain Tumor MRI dataset (Table 1), whereas the few-shot experiment uses an Brain Tumor MRI Dataset [31]. These datasets differ in sample size, class composition, and imaging protocol. Furthermore, few-shot fine-tuning with extremely limited labeled samples (1–32 per class) may induce catastrophic forgetting of the pretrained cross-modal alignment, particularly when the target domain exhibits a non-negligible distribution shift relative to the pretraining corpus. Consequently, the observed accuracy gap reflects dataset heterogeneity and fine-tuning instability rather than an intrinsic reversal of task difficulty.

### Visual question answering

In the design of the medical VQA experiments, we aimed to align the evaluation scenario closely with the core objective of this study: multimodal analysis of cancer pathology. To avoid interference from non-cancer-related pathology questions, we conducted a targeted filtering process on the original question sets of the selected datasets.

The filtering criteria were based on the core terminology lexicon of cancer pathology developed in this study. For each original question, we performed keyword matching and semantic relevance assessment, retaining only questions that met the requirements of cancer pathology diagnostic reasoning.

**Figure 4:**
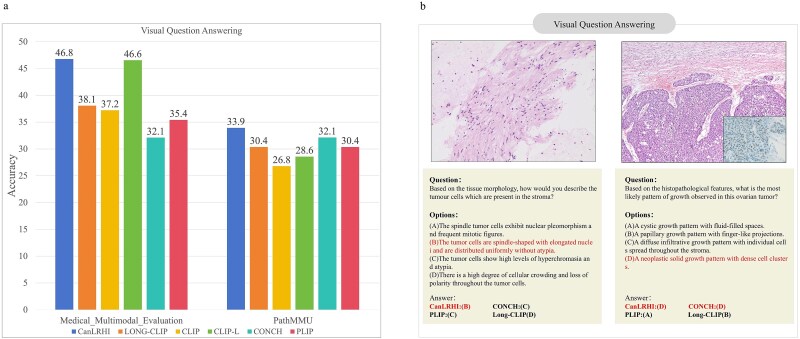
Results of the VQA task. (a) Performance of CanLRHI, CONCH, and other models on the medical multimodal evaluation and PathMMU datasets, evaluated by accuracy, highlighting differences in model performance for medical VQA tasks. (b) Two representative question–answer examples from the PathMMU dataset.

Experimental results show that CanLRHI achieved a significantly higher overall accuracy than the comparison models in cancer pathology VQA tasks. As shown in Fig. 4, on the Medical Multimodal Evaluation dataset [32], CanLRHI reached an accuracy of 46.8%, representing an 11.4% improvement over CONCH (35.4%). On the PathMMU dataset [33], the model achieved 46.6%, outperforming CONCH (32.1%) by 14.5%. Its advantage is evident not only in answering general pathological questions but also in those involving tumor subtyping, grading, and infiltration characteristics, where CanLRHI demonstrates superior accuracy and adaptability in cross-modal reasoning. Similar to zero-shot classification, VQA evaluation follows the standard protocol of the original benchmarks: the model is directly applied to the held-out test sets of Medical Multimodal Evaluation and PathMMU without any task-specific training or cross-validation. This protocol is consistent with the established evaluation conventions of both datasets and ensures comparability with prior work.

### Cross-modal retrieval

The cross-modal retrieval task serves as a core experimental setting for evaluating the semantic alignment accuracy between pathological images and long pathological reports. Specifically, its performance directly reflects the model’s capability for bidirectional mapping between visual and textual modalities. This task is of great clinical significance, supporting applications such as similar case retrieval, image-report verification, and automated pathology report matching.

**Figure 5:**
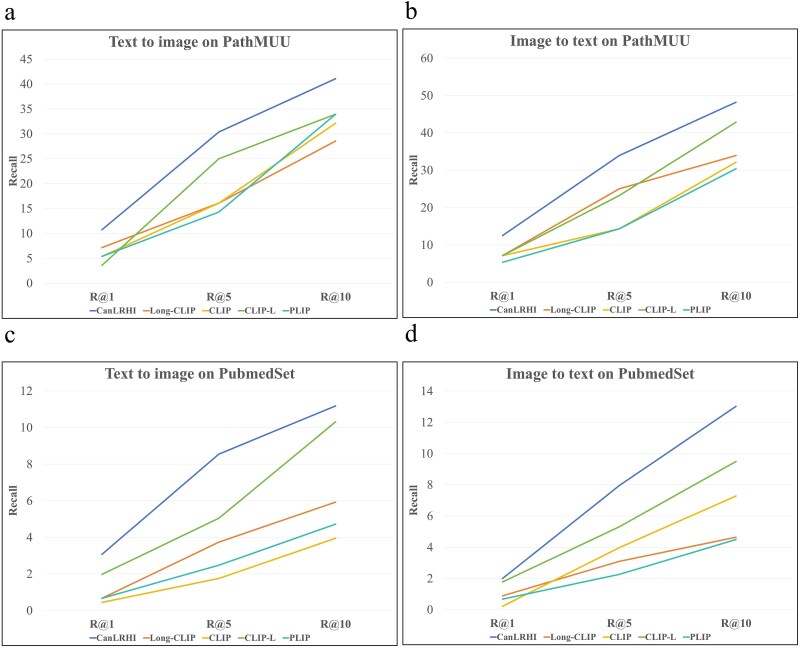
Cross-modal retrieval performance of the CanLRHI model on the PathMMU and PubMedSet pathology datasets.

To comprehensively verify the cross-modal alignment ability of CanLRHI in cancer pathology scenarios, experiments were conducted on two public pathology datasets, PathMMU and PubMedSet, involving two retrieval directions: Text-to-Image and Image-to-Text. Evaluation metrics specifically include recall at ranks 1, 5, and 10 (R@1, R@5, and R@10). As illustrated in Fig. 5, CanLRHI significantly outperforms all baseline models in both retrieval directions, consistently demonstrating higher semantic consistency and retrieval precision across datasets. The model effectively captures deep semantic correlations between pathological images and long diagnostic reports, showing excellent cross-modal generalization ability and providing robust support for downstream clinical applications such as case retrieval and diagnostic assistance.

### Ablation experiment

To further validate the contribution of each key component in CanLRHI, we conducted an ablation study by selectively removing individual modules while keeping all others intact. Specifically, ‘without Long-CLIP Optimization’ denotes replacement of the Long-CLIP text encoder with the standard CLIP text encoder, which retains the original 77-token input limit and forces the model to operate under conventional short-text constraints. ‘without ViT Optimization’ indicates reversion to the standard ViT-L/14 configuration with 224 × 224 input resolution and 14 × 14 patch size, removing all resolution and patch refinements introduced in this work. ‘without Cancer-BART’ represents removal of the medical long-text compression stage, where raw pathology reports are directly fed into Long-CLIP without structured compression.

As shown in Table 2, the complete CanLRHI model achieved the best overall performance across five representative cancer pathology datasets, with an average accuracy of 61.57%. When the Long-CLIP text encoder optimization was removed, model performance dropped significantly (average accuracy decreased to 46.52%), with a particularly sharp decline to 17.49% on the Malignant Lymphoma Classification dataset. This indicates that the long-text encoding mechanism plays a crucial role in capturing fine-grained diagnostic details within pathology reports. Similarly, removing the ViT optimization led to a marked performance degradation (average accuracy 44.80%), with substantial declines on Brain Tumor MRI (20.43%) and Br35H (53.33%), underscoring the importance of high-resolution visual feature extraction for cancer pathology image analysis. The ‘without Cancer-BART’ condition further highlights the necessity of domain-specific text compression for preserving diagnostic semantics; without structured compression, raw reports may exceed token limits or introduce redundant background noise that impairs alignment precision.

**Table 2:** Ablation study results of CanLRHI on five representative cancer pathology datasets.

**Model variant**	**Brain Tumor MRI**	**Chest CT-Scan**	**Malignant lymphoma classification**	**Dataset_BUSI_with_GT**	**Br35H**	**Average**
CanLRHI (Full Model)	65.81	51.32	65.03	38.22	87.47	61.57
without Long-CLIP Optimization	48.07	49.03	17.49	36.26	81.73	46.52
without ViT Optimization	20.43	51.05	64.48	34.72	53.33	44.80
without Cancer-BART	23.96	31.61	33.63	32.19	50	34.28

In summary, Long-CLIP text encoder optimization, ViT visual encoder optimization, and Cancer-BART medical compression are all essential components for ensuring robust cross-modal alignment and achieving superior performance in medical zero-shot classification tasks.

## Discussion

In this study, we constructed a specialized multimodal dataset for cancer pathology, named CancerPath-170 K-v1. This dataset provides a solid foundation for both model training and evaluation, and it offers high-quality image-text paired resources for investigating the correlation between cancer region morphological features and cell death regulatory mechanisms in oncology research. Addressing the limitations of existing multimodal models in handling long medical texts, CanLRHI introduces systematic improvements at the text-encoding level. This represents the core innovation of our work, enabling accurate extraction of cancer pathological semantic features closely associated with cell death status and tumor microenvironment characteristics.

Specifically, CanLRHI employs the Cancer-BART model, fine-tuned on medical corpora, to perform structured compression of lengthy pathology reports. This approach effectively removes non-diagnostic redundancy while preserving key semantic elements such as lesion type, grading, and infiltration extent -core pathological indicators that directly reflect the cell death propensity and invasive characteristics of cancer cells [34]. The compressed text is then encoded using the Long-CLIP encoder, which supports long-sequence modeling, thereby overcoming the short-text limitation of conventional CLIP and enabling faithful semantic representation and cross-modal alignment of complete pathology information. This ‘compression-expansion dual-stage modeling mechanism’ significantly enhances semantic completeness and alignment precision in long-text scenarios, laying a robust semantic foundation for the multimodal characterization of cancer regions in cell death-related cancer pathology research.

Crucially, the cancer region identification capability demonstrated by CanLRHI across all downstream tasks directly supports cell death analysis. In cancer pathology, cell death regulatory mechanisms—including apoptosis, necrosis, and ferroptosis—exhibit significant spatial heterogeneity within tumor tissues. Accurate identification of cancer regions through multimodal alignment enables subsequent localization of cell death hotspots, correlation with histological grading, and extraction of biomarker-related morphological features. For instance, in the zero-shot classification experiments, CanLRHI’s ability to distinguish malignant from benign lesions (e.g. 87.47% accuracy on Br35H) reflects its capacity to capture cancer-specific visual patterns that are intrinsically linked to aberrant cell death regulation. Similarly, in cross-modal retrieval, the alignment between high-resolution pathological images and compressed diagnostic reports preserves semantic information about necrotic regions and apoptotic indices, providing a multimodal foundation for quantitative cell death studies.

On the visual side, optimization is achieved through the integration of a high-resolution ViT architecture and Z-score normalization, which strengthen the model’s ability to capture fine-grained structural details within pathological slides and further improve the accurate identification and fine-grained representation of cancer regions. Together, these components allow CanLRHI to fully integrate textual and visual information within a unified multimodal semantic space, aligning with recent advances in pathology vision-language models.

Through its long-text-driven semantic compression and alignment mechanism [35], the model provides a novel and generalizable framework for multimodal representation learning in cancer pathology and aligns with emerging medical foundation models such as Med-Flamingo [36]. This work not only establishes the foundation for more precise diagnostic modeling and clinical decision support in oncology, but also offers a reliable technical tool for exploring the regulatory mechanisms of cell death in cancer and analyzing the correlation between cancer subtypes and cell death modes [37, 38].

**Key Points**

- We propose the Cancer-BART model fine-tuned on medical corpora to achieve structured compression of pathological long texts, which fully preserves core diagnostic information while satisfying model input constraints and avoids semantic loss.
- The Long-CLIP encoder is adopted to break through the input length limitation of conventional CLIP, so as to adapt to the complete encoding requirements of the compressed long texts.
- We deeply fuse complete pathological diagnostic content in long texts with visual features, and enhance cross-modal semantic alignment through symmetric contrastive learning, further solving the problem of insufficient alignment accuracy caused by the lack of diagnostic context in short-text modeling.

## Data Availability

The datasets and codes are available at https://github.com/nefuWL/CanLRHI.

## References

[1] Allison KH, Sledge GW. Heterogeneity and cancer. *Oncology* 2014;28:772–2, 8. 25224475

[2] Madabhushi A, Lee G. Image analysis and machine learning in digital pathology: challenges and opportunities. *Med Image Anal* 2016;33:170–5. 10.1016/j.media.2016.06.037 27423409 PMC5556681

[3] Simpson MS, Demner-Fushman D. Biomedical text mining: A survey of recent progress. In: Aggarwal C, Zhai C. (eds), *Mining Text Data*, pp. 465–517. Boston, MA: Springer, 2012. 10.1007/978-1-4614-3223-4_14.

[4] Radford A, Kim JW, Hallacy C et al. Learning transferable visual models from natural language supervision. In: Marina Meila, Tong Zhang (eds), International Conference on Machine Learning, pp. 8748–63. Vienna, Austria: PmLR, 2021.

[5] Li J, Selvaraju R, Gotmare A et al. Align before fuse: vision and language representation learning with momentum distillation. *Adv Neural Inf Proces Syst* 2021;34:9694–705.

[6] Li J, Li D, Xiong C et al. Blip: Bootstrapping language-image pre-training for unified vision-language understanding and generation. In: Chaudhuri K, Jegelka S, Song L, Szepesvari C (eds), International Conference on Machine Learning, pp. 12888–900. Baltimore, MD, USA: PMLR, 2022.

[7] Wang Z, Wu Z, Agarwal D et al. Medclip: Contrastive learning from unpaired medical images and text. In: Goldberg Y, Kozareva Z, Zhang Y (eds), Proceedings of the 2022 Conference on Empirical Methods in Natural Language Processing, pp. 3876–87, Abu Dhabi, United Arab Emirates: Association for Computational Linguistics, 2022. 10.18653/v1/2022.emnlp-main.256 PMC11323634 39144675

[8] Lewis M, Liu Y, Goyal N et al. BART: Denoising sequence-to-sequence pre-training for natural language generation, translation, and comprehension. In: Chai J, Schluter N, Tetreault J (eds), Proceedings of the 58th Annual Meeting of the Association for Computational Linguistics, pp. 7871–80, Seattle, WA, USA: Association for Computational Linguistics, 2020.

[9] Zhang B, Zhang P, Dong X et al. Long-clip: Unlocking the long-text capability of clip. In: Leonardis A, Ricci E, Roth S, Russakovsky O, Sattler T, Varol G (eds), *European Conference on Computer Vision*, pp. 310–25. Cham, Milan, Italy: Springer, 2024. 10.1007/978-3-031-72983-6_18.

[10] Wang W, Zheng VW, Yu H et al. A survey of zero-shot learning: settings, methods, and applications. *ACM Trans Intell Syst Technol* 2019;10:1–37. 10.1145/3293318

[11] Xian Y, Schiele B, Akata Z. Zero-shot learning-the good, the bad and the ugly. In: Rehg JM, Lazebnik S (eds), Proceedings of the IEEE Conference on Computer Vision and Pattern Recognition, pp. 4582–91, Honolulu, HI, USA: IEEE, 2017.

[12] Liu H, Tam D, Muqeeth M et al. Few-shot parameter-efficient fine-tuning is better and cheaper than in-context learning. *Adv Neural Inf Proces Syst* 2022;35:1950–65.

[13] Antol S, Agrawal A, Lu J et al. Vqa: Visual question answering. In: Ikeuchi K, Schnörr C, Sivic J, Vidal R (eds) Proceedings of the IEEE international conference on computer vision, pp. 2425–33, Santiago, Chile: IEEE, 2015.

[14] Wang T, Li F, Zhu L et al. Cross-modal retrieval: a systematic review of methods and future directions. *Proc IEEE* 2025;112:1716–54. 10.1109/JPROC.2024.3525147

[15] Lin W, Zhao Z, Zhang X et al. Pmc-clip: Contrastive language-image pre-training using biomedical documents. In: Greenspan H, Madabhushi A, Mousavi P et al. (eds), International Conference on Medical Image Computing and Computer-Assisted Intervention, pp. 525–36. Cham, Vancouver, BC, Canada: Springer, 2023. 10.1007/978-3-031-43993-3_51.

[16] Bodenreider O . The unified medical language system (UMLS): integrating biomedical terminology. *Nucleic Acids Res* 2004;32:267D–0. 10.1093/nar/gkh061 PMC308795 14681409

[17] Sedgwick P . Standardising outcome measures using z scores. *BMJ* 2014;349:g5878. 10.1136/bmj.g5878 25273560

[18] Dosovitskiy A, Beyer L, Kolesnikov A et al. An image is worth 16x16 words: transformers for image recognition at scale. In: Mohamed S, Hofmann K, Murray N, Oh A, Titov I (eds), International Conference on Learning Representations, 2021. OpenReview.net, Vienna, Austria, 2021.

[19] Chen T, Kornblith S, Norouzi M et al. A simple framework for contrastive learning of visual representations. In: Daumé III H, Singh A (eds), *International Conference on Machine Learning*, pp. 1597–607. Vienna, Austria: PmLR, 2020.

[20] Gower RM, Loizou N, Qian X et al. SGD: General analysis and improved rates. In: Chaudhuri K, Salakhutdinov R (eds), *International Conference on Machine Learning*, pp. 5200–9. Long Beach, CA, USA: PMLR, 2019.

[21] Huang M, Yang W, Wu Y et al. Brain tumor segmentation based on local independent projection-based classification. *IEEE Trans Biomed Eng* 2014;61:2633–45. 10.1109/TBME.2014.2325410 24860022

[22] Syed SA, Rehan, Iqbal A. Medical Imagining (CT Scan, MRI, X-Ray, and Microscopic Imagery) Data 2024. Mendeley Data, V1, 10.17632/5kbjrgsncf.1

[23] Orlov NV, Chen WW, Eckley DM et al. Automatic classification of lymphoma images with transform-based global features. *IEEE Trans Inf Technol Biomed* 2010;14:1003–13. 10.1109/TITB.2010.2050695 20659835 PMC2911652

[24] Al-Dhabyani W, Gomaa M, Khaled H et al. Dataset of breast ultrasound images. *Data in brief* 2020;28:104863. 10.1016/j.dib.2019.104863 31867417 PMC6906728

[25] Hossain T, Shishir FS, Ashraf M et al. Brain tumor detection using convolutional neural network. In: Piuri V, Reza AW (eds), 2019 1st International Conference on Advances in Science, Engineering and Robotics Technology (ICASERT), pp. 1–6. Dhaka, Bangladesh: IEEE, 2019.

[26] Lu MY, Chen B, Williamson DF et al. A visual-language foundation model for computational pathology. *Nat Med* 2024;30:863–74. 10.1038/s41591-024-02856-4 38504017 PMC11384335

[27] Huang Z, Bianchi F, Yuksekgonul M et al. A visual–language foundation model for pathology image analysis using medical twitter. *Nat Med* 2023;29:2307–16. 10.1038/s41591-023-02504-3 37592105

[28] Snell J, Swersky K, Zemel R. Prototypical networks for few-shot learning. *Adv Neural Inf Proces Syst* 2017;30:4077–4087.

[29] Finn C, Abbeel P, Levine S. Model-agnostic meta-learning for fast adaptation of deep networks. In: Precup D, Teh YW (eds), International Conference on Machine Learning, pp. 1126–35. Sydney, Australia: PMLR, 2017.

[30] Esteva A, Kuprel B, Novoa RA et al. Dermatologist-level classification of skin cancer with deep neural networks. *nature* 2017;542:115–8. 10.1038/nature21056 28117445 PMC8382232

[31] M Nickparvar . Brain Tumor MRI Dataset. 2026.

[32] Chen J, Gui C, Ouyang R et al. Towards injecting medical visual knowledge into multimodal llms at scale. In: Al-Onaizan Y, Bansal M, Chen YN (eds), Proceedings of the 2024 Conference on Empirical Methods in Natural Language Processing, pp. 7346–70, Miami, Florida, USA: Association for Computational Linguistics, 2024.

[33] Sun Y, Wu H, Zhu C et al. Pathmmu: A massive multimodal expert-level benchmark for understanding and reasoning in pathology. In: Leonardis A, Ricci E, Roth S, Russakovsky O, Sattler T, Varol G (eds), *European Conference on Computer Vision*, pp. 56–73. Cham: Springer, 2024. 10.1007/978-3-031-73033-7_4

[34] Galluzzi L, Maiuri MC, Vitale I et al. Cell death modalities: classification and pathophysiological implications. *Cell Death & Differentiation,* 2007;14:1237–43. 10.1038/sj.cdd.4402148 17431418

[35] Liu H, Li C, Wu Q et al. Visual instruction tuning. *Advances in neural information processing systems, Advances in neural information processing systems* 2023;36:41076–258.38505104

[36] Moor M, Huang Q, Wu S et al. Med-flamingo: A multimodal medical few-shot learner. In: Hegselmann S, Parziale A, Shanmugam D et al. (eds), *Machine Learning for Health (ML4H)*, pp. 353–67. New Orleans, LA, USA: PMLR, 2023.

[37] Koren E, Fuchs Y. Modes of regulated cell death in cancer. *Cancer Discov* 2021;11:245–65. 10.1158/2159-8290.CD-20-0789 33462123

[38] Hu X-m, Li Z-x, Lin R-h et al. Guidelines for regulated cell death assays: a systematic summary, a categorical comparison, a prospective. *Front Cell Dev Biol* 2021;9:634690. 10.3389/fcell.2021.634690 33748119 PMC7970050

